# Heat Stress Responses During Grain Filling in Two Korean Wheat Cultivars as Affected by Developmental Stage and Exposure Duration

**DOI:** 10.3390/plants15203130

**Published:** 2026-10-13

**Authors:** Ju-Young Choi, Junseok Choi, Jae-Han Son, Yurim Kim, Han-Yong Jeong, Jinhee Park, Goeun Lee, Sookjin Kim, Chon-Sik Kang, Chuloh Cho

**Affiliations:** Winter Crop Research Division, National Institute of Crop and Food Science, Rural Development Administration, Wanju 55365, Republic of Korea; jychoi8519@gmail.com (J.-Y.C.);

**Keywords:** endosperm development, gene expression, protein concentration, RNA-seq, starch metabolism

## Abstract

High temperature can impair grain development and storage compound accumulation during wheat grain filling, with responses depending on stress intensity, timing, duration, and cultivar. In this study, Saekeumkang and Hwanggeumal were selected from among major Korean wheat cultivars because they differ in grain quality- and starch-related characteristics, providing a basis for comparing cultivar-specific responses to heat stress during grain filling. Plants were exposed to 25/15, 35/25, and 40/30 °C (day/night) temperature regimes for 5 d beginning at 10 or 20 days after heading (DAH), and changes in grain morphology, endosperm and embryo development, 100-grain weight, protein concentration, and storage-related gene expression were evaluated. High temperature altered grain developmental traits and generally reduced 100-grain weight, while the magnitude of these effects differed between cultivars and treatment stages. Grain protein concentration and the expression of starch- and storage protein-related genes also showed cultivar- and stage-dependent responses. In a separate RNA-seq experiment initiated at 10 DAH, plants were exposed to the temperature treatments for either 5 or 10 d, and transcriptomic analysis revealed that heat intensity and exposure duration affected both the number and functional characteristics of differentially expressed genes, particularly those associated with protein folding and quality control, chromatin-related processes, and carbohydrate metabolism. These results indicate that heat responses during wheat grain filling are jointly influenced by developmental stage, exposure duration, and cultivar-specific molecular regulation.

## 1. Introduction

Wheat (*Triticum aestivum* L.) is one of the most important cereal crops worldwide, and its grain yield and quality are greatly affected by environmental conditions, particularly temperature during reproductive development and grain filling. During this period, assimilates are transported to developing grains and accumulate mainly in the form of starch and storage proteins, ultimately determining grain size, weight, and quality. Heat stress during grain filling accelerates crop development, shortens the effective grain filling period, and limits dry matter accumulation in developing grains, thereby reducing grain weight and yield [1,2,3,4]. Severe high temperatures during grain development can accelerate the attainment of maximum grain dry weight [5], and elevated temperatures significantly shorten the starch accumulation period in developing wheat grains [6]. Furthermore, studies on Korean wheat cultivars have revealed that responses to elevated temperatures during grain filling differ among genotypes, thereby affecting grain growth, yield, flour characteristics, and quality traits [1,2,7]. Therefore, understanding wheat responses to heat stress during grain filling is becoming increasingly important for stable wheat production under warming environments and for identifying genetic resources that are better adapted to high temperatures.

The effects of heat stress on developing wheat grains are not uniform throughout the grain filling period. The physiological and metabolic status of developing grains continuously changes during grain filling; therefore, the effects of heat exposure can be strongly influenced by the developmental stage at which stress occurs [1,8,9]. Heat stress experienced during the early grain filling period may have persistent effects on subsequent grain development and the accumulation of storage compounds, whereas stress occurring at later stages affects grains that have already accumulated a considerable proportion of their storage reserves. The degree of reduction in grain size and weight caused by heat also varies depending on the intensity and duration of heat exposure, as well as the genotype [1,3,4,8]. In addition, heat stress can accelerate leaf senescence and inhibit photosynthetic carbon assimilation, thereby limiting the supply of assimilates to developing grains [4]. Taken together, these findings indicate that both the timing and intensity of heat exposure have important effects on grain filling. Therefore, evaluating developmental responses to heat stress at different stages of grain filling is essential for identifying critical periods of heat sensitivity and understanding differences in heat responses among cultivars.

Final grain weight and quality are closely related to the synthesis and accumulation of starch and storage proteins in the developing endosperm. Starch is the major storage component of wheat grains, and its biosynthesis involves several enzymes, including soluble starch synthase (SS), granule-bound starch synthase (GBSS), and starch branching enzyme (SBE). The biosynthetic pathways associated with these enzymes are sensitive to elevated temperatures [6,10]. In particular, high temperatures can reduce the activity of soluble starch synthase, thereby decreasing starch accumulation in wheat endosperm [10]. Under high temperature conditions, the starch accumulation period is shortened, and the expression of genes encoding major enzymes involved in starch biosynthesis is altered [6]. Reduced starch accumulation can limit dry matter deposition in developing grains, which may ultimately decrease final grain weight. Heat stress during grain filling also alters the concentration and composition of storage proteins, including gliadins and glutenins, and affects the expression of genes involved in storage protein synthesis and heat stress responses [3,7,9]. Consistent with these changes, heat stress has been reported to alter grain protein concentration while reducing grain weight and the duration of grain filling [3].

Heat responses in wheat are coordinated across physiological, biochemical, and molecular levels. Physiological changes such as altered photosynthetic carbon supply and accelerated senescence are accompanied by changes in storage-compound metabolism and by transcriptional regulation of stress-responsive and metabolic genes [4,6,8,9].

At the molecular level, heat stress induces extensive changes in gene expression and metabolic regulation during grain development. Transcriptome analyses of wheat grains have revealed genotype-specific responses to high temperatures, including differential regulation of genes involved in transcription, RNA and protein synthesis, metabolism, and heat stress responses, such as those encoding heat shock proteins [8]. Gene expression and proteomic analyses have also revealed that heat stress during grain filling affects multiple pathways related to starch biosynthesis, storage protein metabolism, transcriptional regulation, stress responses, and primary metabolism [9]. These findings suggest that differences in grain development among cultivars under heat stress may be associated not only with differences in grain morphology and storage compound accumulation but also with distinct transcriptional responses. However, relatively limited information is available on how the timing and intensity of heat stress interact with genotypes to affect grain development and storage metabolism in Korean wheat cultivars, and whether cultivar-specific phenotypic responses are associated with distinct molecular and transcriptomic responses.

In this study, we investigated stage-dependent responses to heat stress during grain filling in two Korean wheat cultivars, Saekeumkang and Hwanggeumal. We hypothesized that the effects of heat stress on grain development and storage metabolism would vary depending on the developmental stage at which the stress was imposed and that these responses would differ between cultivars. Phenotypic, grain-quality, gene-expression, and transcriptomic analyses were integrated to characterize stage- and cultivar-specific responses to high temperature. This study provides fundamental information for evaluating heat responses in Korean wheat cultivars and for supporting stable wheat production and cultivar improvement under increasingly high-temperature environments.

## 2. Results

### 2.1. Changes in Grain Morphology Under High-Temperature Treatment at Different Grain-Filling Stages

**Figure 1:**
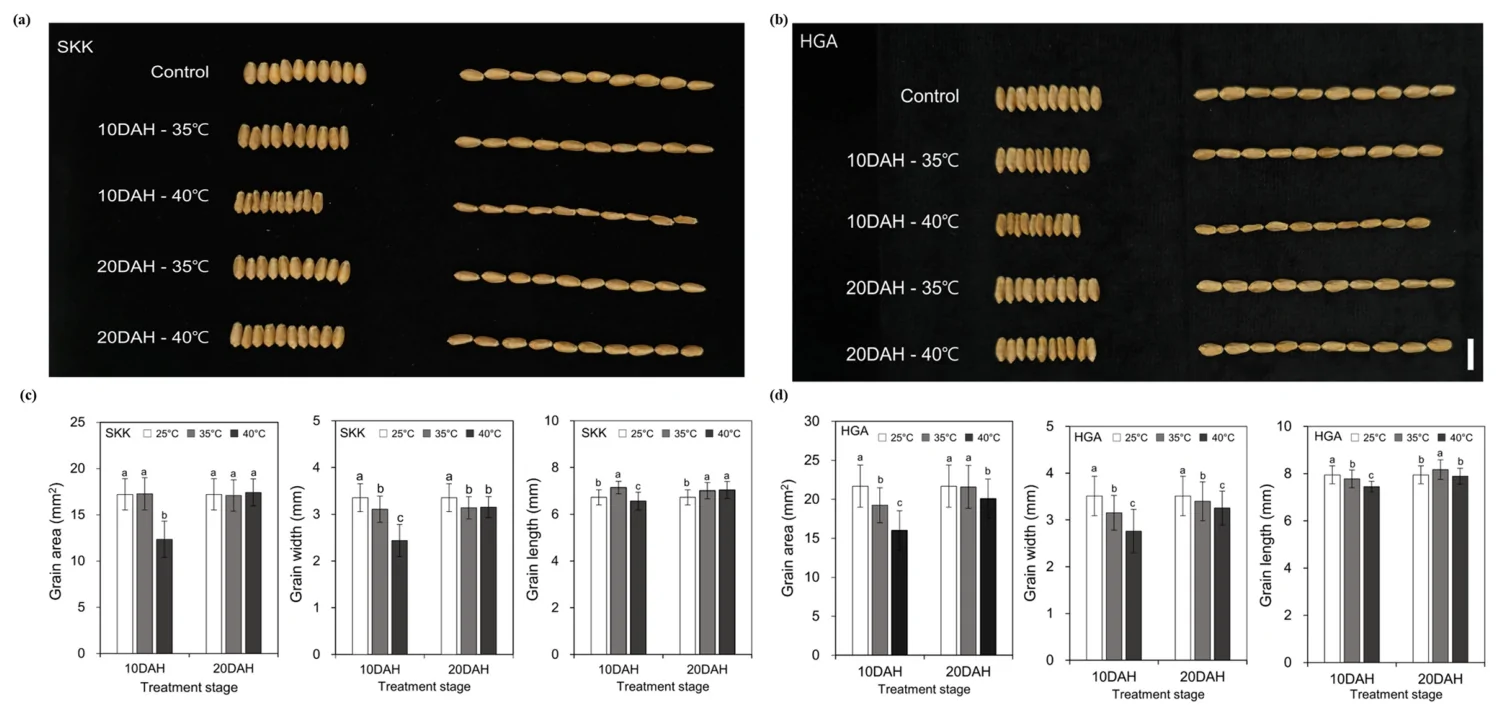
Effects of heat treatment at different grain-filling stages on grain morphology in Saekeumkang (SKK) and Hwanggeumal (HGA). (**a**) Representative grain images of SKK and (**b**) HGA under the control and heat treatments initiated at 10 and 20 days after heading (DAH). (**c**) Grain area, grain length, and grain width of SKK and (**d**) HGA under the respective treatment conditions. The white scale bar indicates 1 cm. Different lowercase letters indicate significant differences among treatments (*p* < 0.05) according to Duncan’s multiple range test.

To determine the effects of high-temperature treatment at different grain-filling stages on grain development, the grain area, length, and width of Saekeumkang and Hwanggeumal were investigated. When high-temperature treatment was initiated at 10 days after heading (10 DAH), both cultivars showed reductions in grain area, length, and width at 35 and 40 °C compared with the control, and these reductions were more pronounced at 40 °C (Figure 1).

When high-temperature treatment was initiated at 20 days after heading (20 DAH), changes in grain morphology were also observed, but the responses varied depending on the cultivar and trait. In Saekeumkang, grain length and width decreased under high-temperature treatment, whereas changes in grain area were relatively small. In Hwanggeumal, grain length and width also changed according to treatment temperature, while grain area tended to decrease at 40 °C (Figure 1).

### 2.2. Changes in Grain Morphology and Endosperm and Embryo Development Under High-Temperature Treatment at Different Grain-Filling Stages

**Figure 2:**
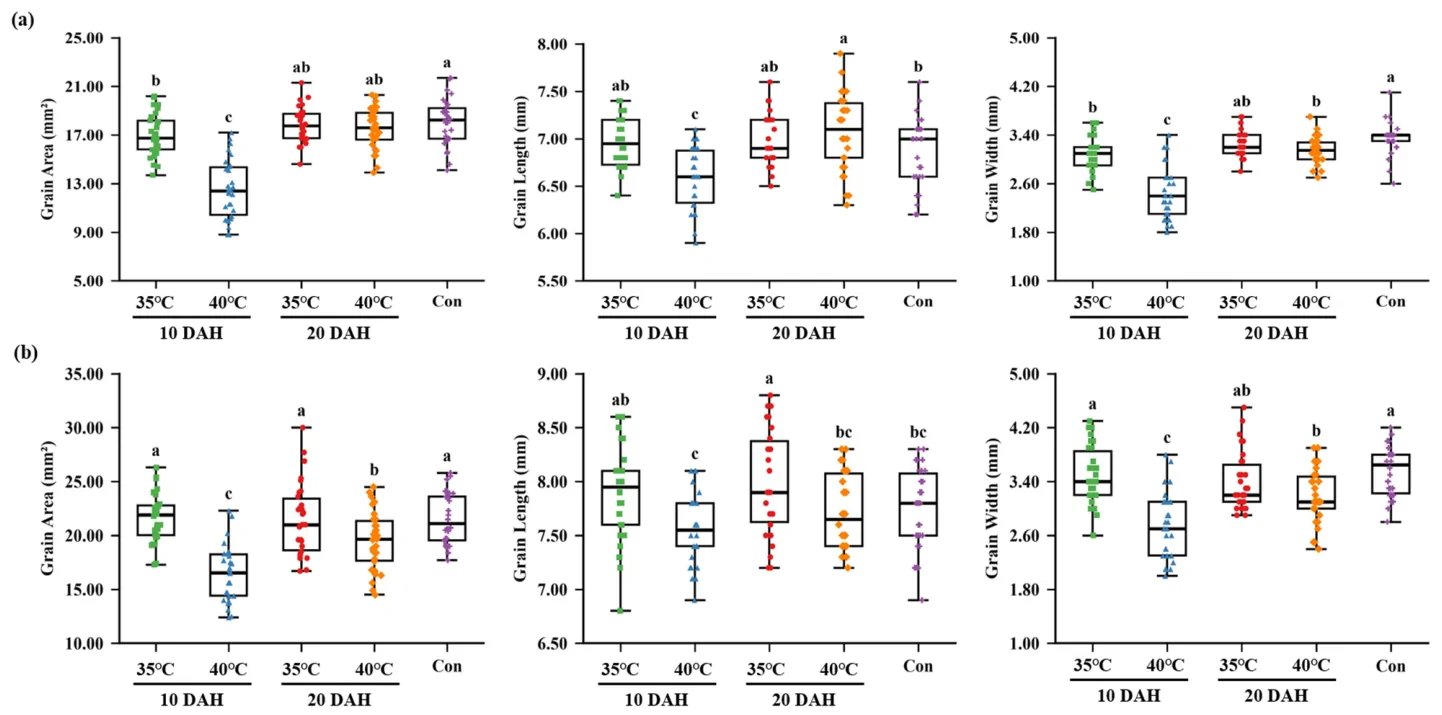
Changes in grain morphological traits of Saekeumkang (SKK) and Hwanggeumal (HGA) under heat treatment at different grain-filling stages. (**a**) Grain area, grain length, and grain width of SKK. (**b**) Grain area, grain length, and grain width of HGA. Different lowercase letters indicate significant differences among treatments (*p* < 0.05) according to Duncan’s multiple range test. Con represents the control treatment at 25 °C.

In the grain morphology analysis, Saekeumkang showed the lowest grain area, length, and width in the 10 DAH treatment at 40 °C, with significant differences from the control. At 20 DAH, differences in grain area among treatments were relatively small, and grain length in the 40 °C treatment was higher than that in the 10 DAH treatment at 40 °C. In Hwanggeumal, grain area, length, and width decreased in the 10 DAH treatment at 40 °C, and grain area and width were significantly lower than those at 35 °C and in the control. At 20 DAH, grain area and width were lower in the 40 °C treatment than in the 35 °C treatment (Figure 2).

**Figure 3:**
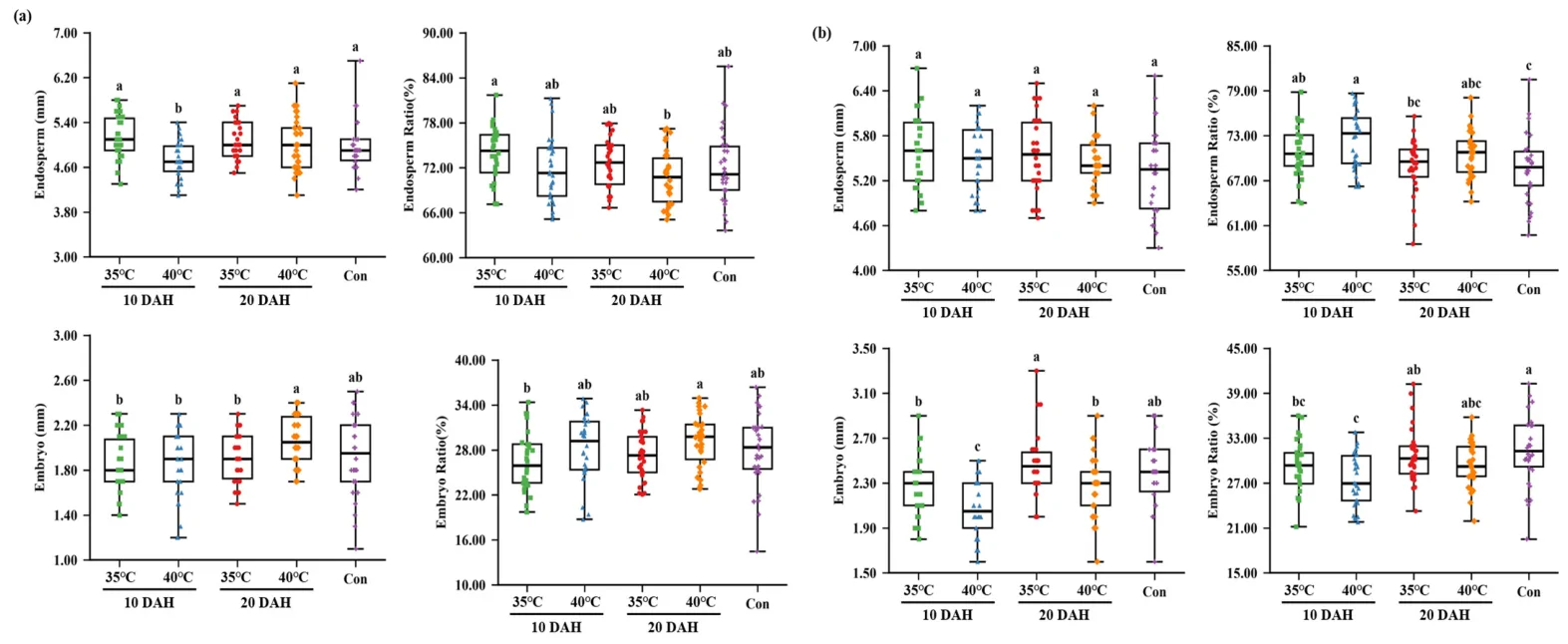
Changes in endosperm and embryo development of Saekeumkang (SKK) and Hwanggeumal (HGA) under heat treatment at different grain-filling stages. (**a**) Endosperm length, endosperm ratio, embryo length, and embryo ratio of SKK. (**b**) Endosperm length, endosperm ratio, embryo length, and embryo ratio of HGA. Different lowercase letters indicate significant differences among treatments (*p* < 0.05) according to Duncan’s multiple range test. Con represents the control treatment at 25 °C.

Analysis of endosperm and embryo development showed that endosperm length in Saekeumkang was significantly lower in the 10 DAH treatment at 40 °C than in the other treatment groups. The endosperm ratio was lower in the 20 DAH treatment at 40 °C than in the 10 DAH treatment at 35 °C, whereas embryo length and embryo ratio showed relatively higher values in the 20 DAH treatment at 40 °C. In Hwanggeumal, there were no significant differences in endosperm length among treatments, whereas the endosperm ratio differed depending on the treatment conditions. Embryo length was higher in the 20 DAH treatment at 35 °C and lower in the 10 DAH treatment at 40 °C, and the embryo ratio also showed the lowest value in the 10 DAH treatment at 40 °C (Figure 3).

**Figure 4:**
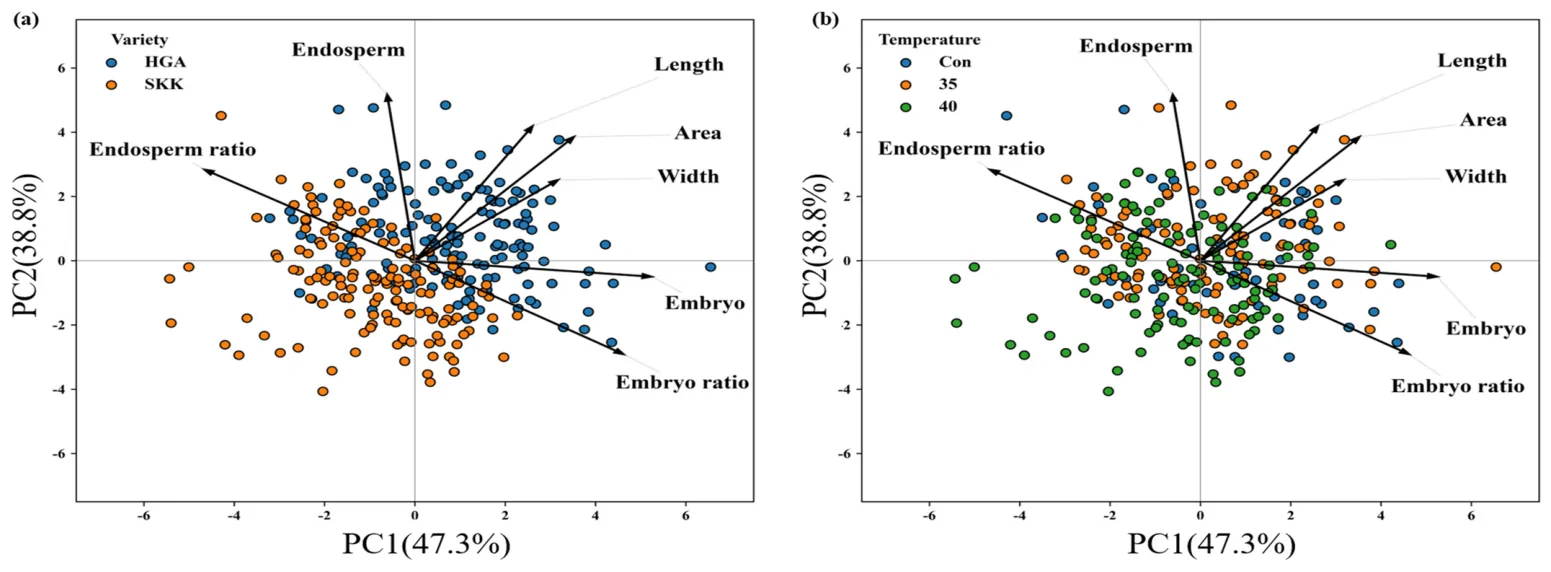
Principal component analysis of grain morphological and internal developmental traits under heat treatment. (**a**) PCA score plot grouped by cultivar (HGA and SKK). (**b**) PCA score plot grouped by temperature treatment (Control, 35 °C, and 40 °C). Vectors indicate the contributions of grain area, grain length, grain width, endosperm length, endosperm ratio, embryo length, and embryo ratio to the principal components. PC1 and PC2 explained 47.3% and 38.8% of the total variance, respectively.

Principal component analysis showed that PC1 and PC2 explained 47.3% and 38.8% of the total variance, respectively, together accounting for 86.1% of the total variance. In the cultivar-based analysis, Saekeumkang and Hwanggeumal showed different distribution patterns along PC1. Grain area, length, and width, as well as embryo-related traits, were mainly positioned in the positive direction of PC1, whereas the endosperm ratio was positioned in the opposite direction. In the temperature-based analysis, some overlap was observed among treatment groups, but differences in distribution along PC1 were observed between the 35 °C and 40 °C treatment groups (Figure 4).

Three-way ANOVA showed significant main effects of cultivar on all grain morphological and internal developmental traits. Significant stage × temperature interactions were detected for grain area, width, and length, while a significant cultivar × stage × temperature interaction was detected only for grain length (Table S1).

### 2.3. Changes in 100-Grain Weight and Protein Concentration Under High-Temperature Treatment at Different Grain-Filling Stages

Analysis of 100-grain weight under high-temperature treatment during grain filling showed that, in Saekeumkang, the 10 DAH treatment at 40 °C resulted in a significantly lower 100-grain weight than the 25 and 35 °C treatments, whereas no significant difference was observed between 25 and 35 °C. At 20 DAH, 100-grain weight was significantly lower at 35 and 40 °C than at 25 °C, while no significant difference was observed between the two high-temperature treatments. In Hwanggeumal, 100-grain weight significantly decreased with increasing treatment temperature at both 10 and 20 DAH, with significant differences among the 25, 35, and 40 °C treatments (Table 1).

Grain protein concentration showed different responses depending on the cultivar and treatment timing. In Saekeumkang, protein concentration significantly increased with increasing treatment temperature at both 10 and 20 DAH, with significant differences among the temperature treatments. In Hwanggeumal, protein concentration at 10 DAH was significantly higher at 40 °C than at 25 °C, whereas the 35 °C treatment did not differ significantly from either 25 or 40 °C. In contrast, at 20 DAH, protein concentration was significantly lower at 35 and 40 °C than at 25 °C, with no significant difference between 35 and 40 °C (Table 1).

Three-way ANOVA showed significant main effects of cultivar on both 100-grain weight and protein concentration. Significant stage × temperature interactions were detected for both traits, whereas the cultivar × temperature interaction was significant only for protein concentration (Table S1).

### 2.4. Temporal Changes in the Expression of Genes Related to Starch and Storage Protein Synthesis Under High-Temperature Treatment

**Figure 5:**
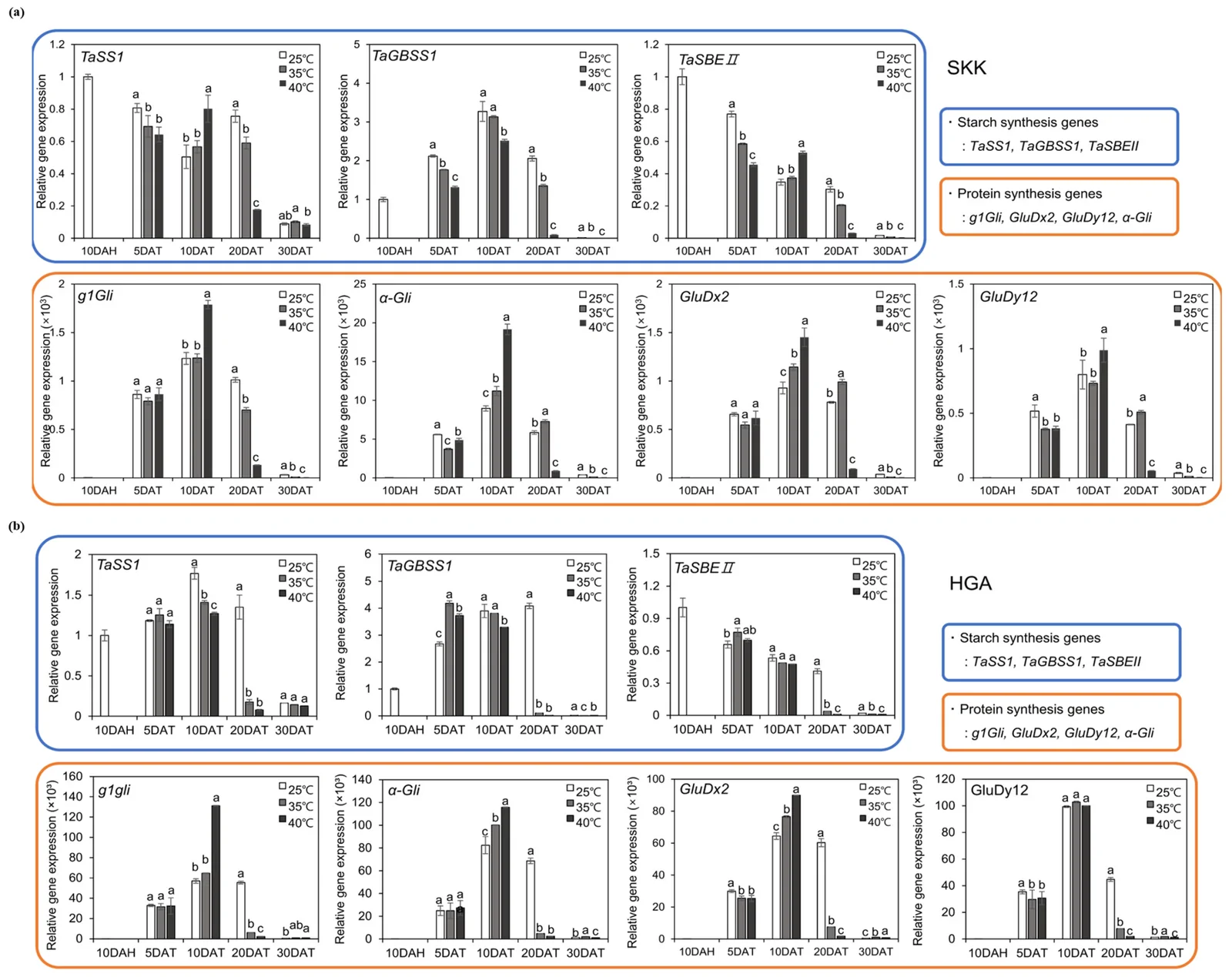
Temporal expression patterns of starch- and storage protein synthesis-related genes in Saekeumkang (SKK) and Hwanggeumal (HGA) under heat treatment initiated at 10 days after heading. (**a**) Relative expression of starch synthesis-related genes (TaSSI, TaGBSSI, and TaSBEII) and storage protein-related genes (g1Gli, α-Gli, Dx2, and Dy12) in SKK. (**b**) Relative expression of the corresponding genes in HGA. Gene expression was analyzed at 10 days after heading (DAH) and at 5, 10, 20, and 30 days after treatment (DAT) under 25, 35, and 40 °C conditions. Different letters indicate significant differences among temperature treatments at each sampling time at *p* < 0.05 by Tukey’s test.

When high-temperature treatment was initiated at 10 DAH, the expression patterns of starch biosynthesis-related and storage protein-related genes varied depending on temperature and sampling time. The starch biosynthesis-related genes TaSSI, TaSBEII, and TaGBSSI generally showed lower expression in plants exposed to 35 and 40 °C than in the 25 °C control at several sampling times, with stronger reductions often observed following the 40 °C treatment. The storage protein-related genes α-Gli, g1Gli, Dx2, and Dy12 showed transient increases at some sampling times following heat treatment, although these responses were not consistently maintained over time (Figure 5).

**Figure 6:**
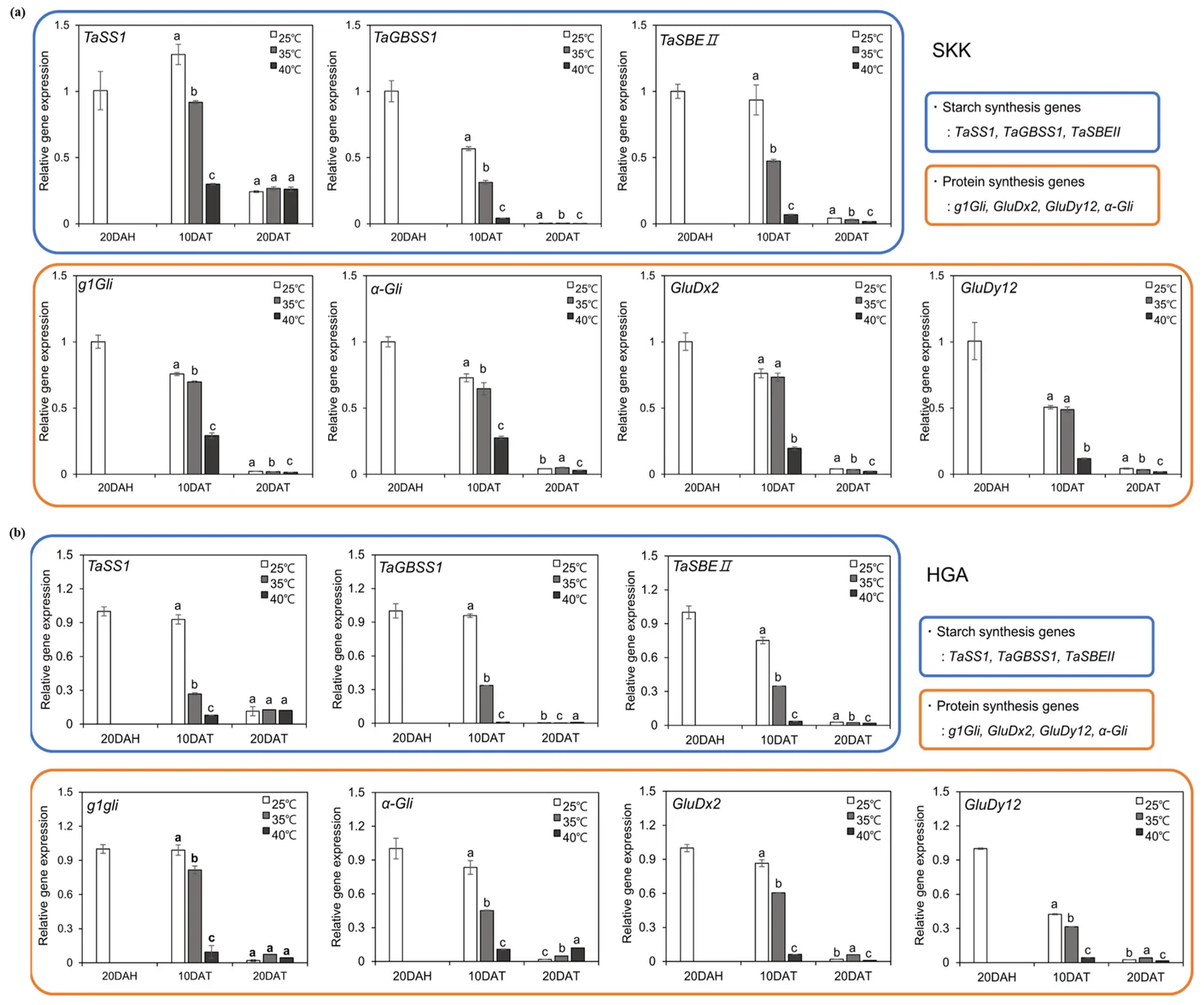
Temporal expression patterns of starch- and storage protein synthesis-related genes in Saekeumkang (SKK) and Hwanggeumal (HGA) under heat treatment initiated at 20 days after heading. (**a**) Relative expression of starch synthesis-related genes (TaSSI, TaGBSSI, and TaSBEII) and storage protein-related genes (g1Gli, α-Gli, Dx2, and Dy12) in SKK. (**b**) Relative expression of the corresponding genes in HGA. Gene expression was analyzed at 20 days after heading (DAH) and at 10 and 20 days after treatment (DAT) under 25, 35, and 40 °C conditions. Different letters indicate significant differences among temperature treatments at each sampling time at *p* < 0.05 by Tukey’s test.

When high-temperature treatment was initiated at 20 DAH, the expression of both starch synthesis-related and storage protein-related genes generally decreased in plants exposed to high temperature compared with the 25 °C control. These reductions were particularly evident at 10 DAT, while expression levels of most genes were low at 20 DAT across temperature treatments (Figure 6). The magnitude of the response varied depending on the gene and cultivar.

### 2.5. Transcriptomic Responses of Saekeumkang and Hwanggeumal to High-Temperature Treatment

**Figure 7:**
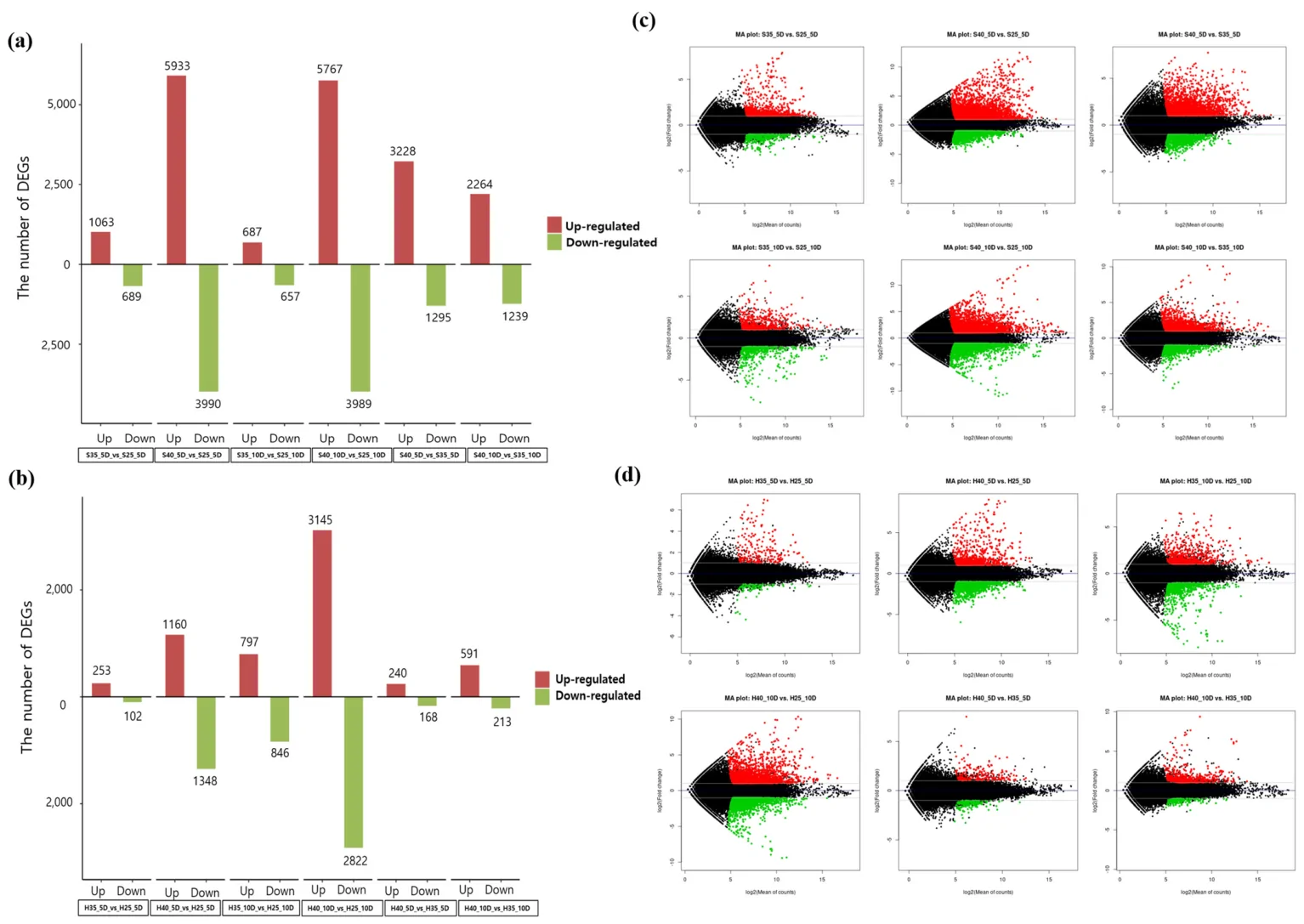
Differentially expressed genes in Saekeumkang (SKK) and Hwanggeumal (HGA) in response to different heat-treatment temperatures and durations. (**a**) Numbers of up- and down-regulated differentially expressed genes (DEGs) in SKK and (**b**) corresponding MA plots for each comparison. (**c**) Numbers of up- and down-regulated DEGs in HGA and (**d**) corresponding MA plots for each comparison. Comparisons included 35 °C versus 25 °C and 40 °C versus 25 °C after 5 and 10 days of treatment, as well as 40 °C versus 35 °C after 5 and 10 days of treatment. In the MA plots, red and green points indicate up- and down-regulated DEGs, respectively, whereas black points indicate non-DEGs. DEGs were defined as genes showing a ≥2-fold change with an FDR-adjusted *p* ≤ 0.01.

RNA-seq analysis showed that in Saekeumkang, after 5 days of treatment, 1063 genes were upregulated and 689 genes were downregulated at 35 °C compared with 25 °C, whereas 5933 and 3990 genes were upregulated and downregulated, respectively, at 40 °C. After 10 days of treatment, 687 upregulated and 657 downregulated DEGs were identified at 35 °C, whereas 5767 upregulated and 3989 downregulated DEGs were identified at 40 °C. In Saekeumkang, more DEGs were identified at 40 °C than at 35 °C after both 5 and 10 days of treatment (Figure 7a).

In Hwanggeumal, 253 upregulated and 102 downregulated DEGs were identified after 5 days of treatment at 35 °C, whereas 1160 and 1348 DEGs were upregulated and downregulated, respectively, at 40 °C. After 10 days of treatment, 797 genes were upregulated and 846 were downregulated at 35 °C, whereas 3145 and 2822 DEGs were upregulated and downregulated, respectively, at 40 °C. In particular, at 40 °C, the number of DEGs in Hwanggeumal was substantially higher after 10 days of treatment than after 5 days (Figure 7c). The corresponding MA plots showed the distribution of up- and down-regulated genes across the different temperature and treatment-duration comparisons in Saekeumkang and Hwanggeumal (Figure 7b,d).

**Figure 8:**
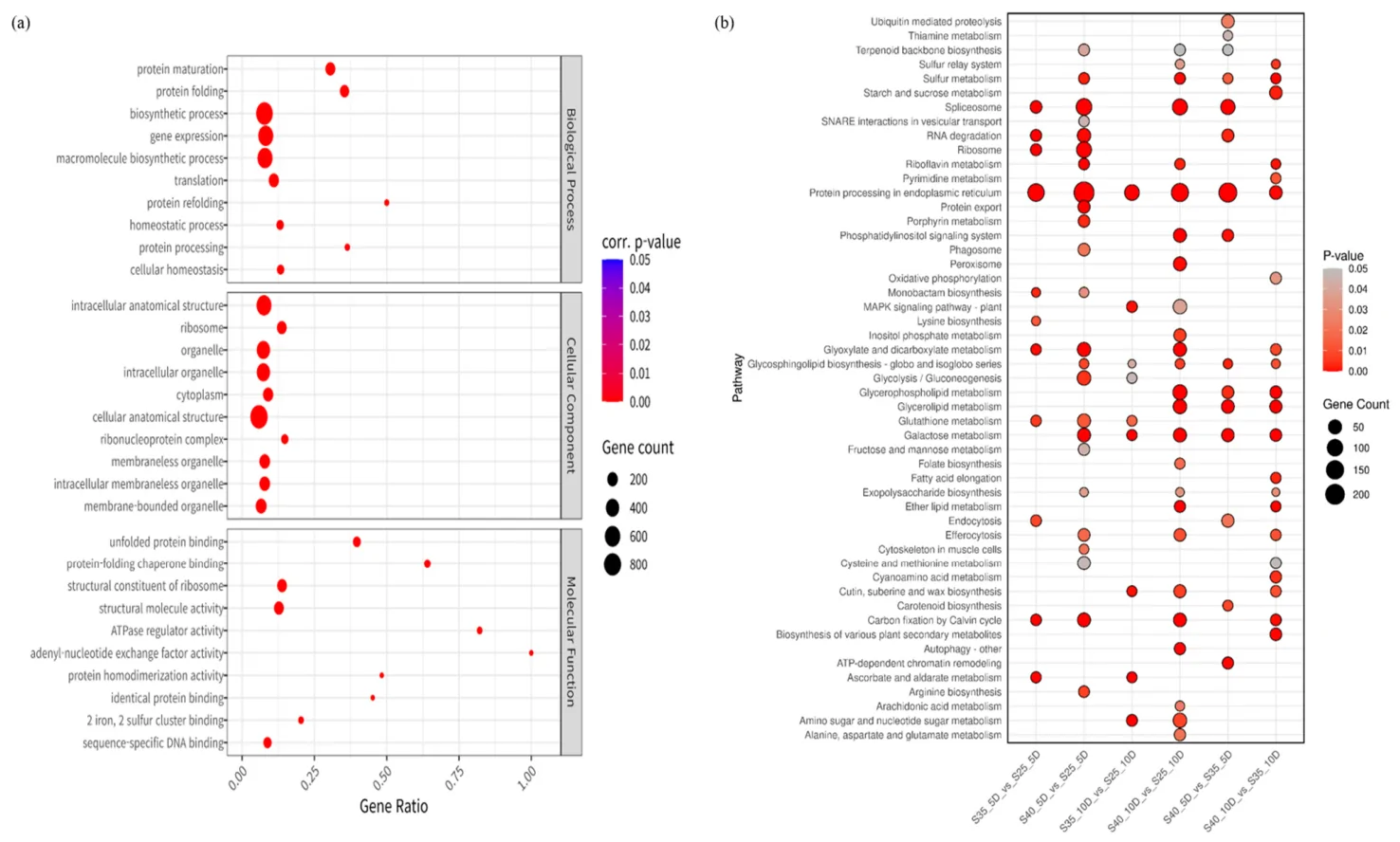
Functional enrichment analysis of up-regulated DEGs in Saekeumkang (SKK) under heat treatment. (**a**) Gene Ontology (GO) enrichment analysis of up-regulated DEGs identified in the S40_5D versus S25_5D comparison. Enriched GO terms are classified into biological process, cellular component, and molecular function categories. The x-axis represents the gene ratio, bubble size indicates the number of genes associated with each GO term, and bubble color represents the corrected *p*-value. (**b**) KEGG pathway enrichment analysis of up-regulated DEGs across temperature and treatment-duration comparisons in SKK. The x-axis represents the comparison groups, the y-axis represents enriched pathways, bubble size indicates the number of genes associated with each pathway, and bubble color represents the *p*-value.

In the GO analysis, upregulated DEGs in Saekeumkang after 5 days of treatment at 40 °C were mainly enriched in categories such as protein maturation, protein folding, gene expression, and translation, while molecular function terms included unfolded protein binding and protein-folding chaperone binding (Figure 8a). KEGG analysis of upregulated DEGs in Saekeumkang showed significant enrichment of protein processing in the endoplasmic reticulum across the high-temperature-versus-control comparisons. RNA degradation was specifically enriched under the 5-day treatment, whereas cutin, suberine and wax biosynthesis were specifically enriched under the 10-day treatment (Figure 8b).

**Figure 9:**
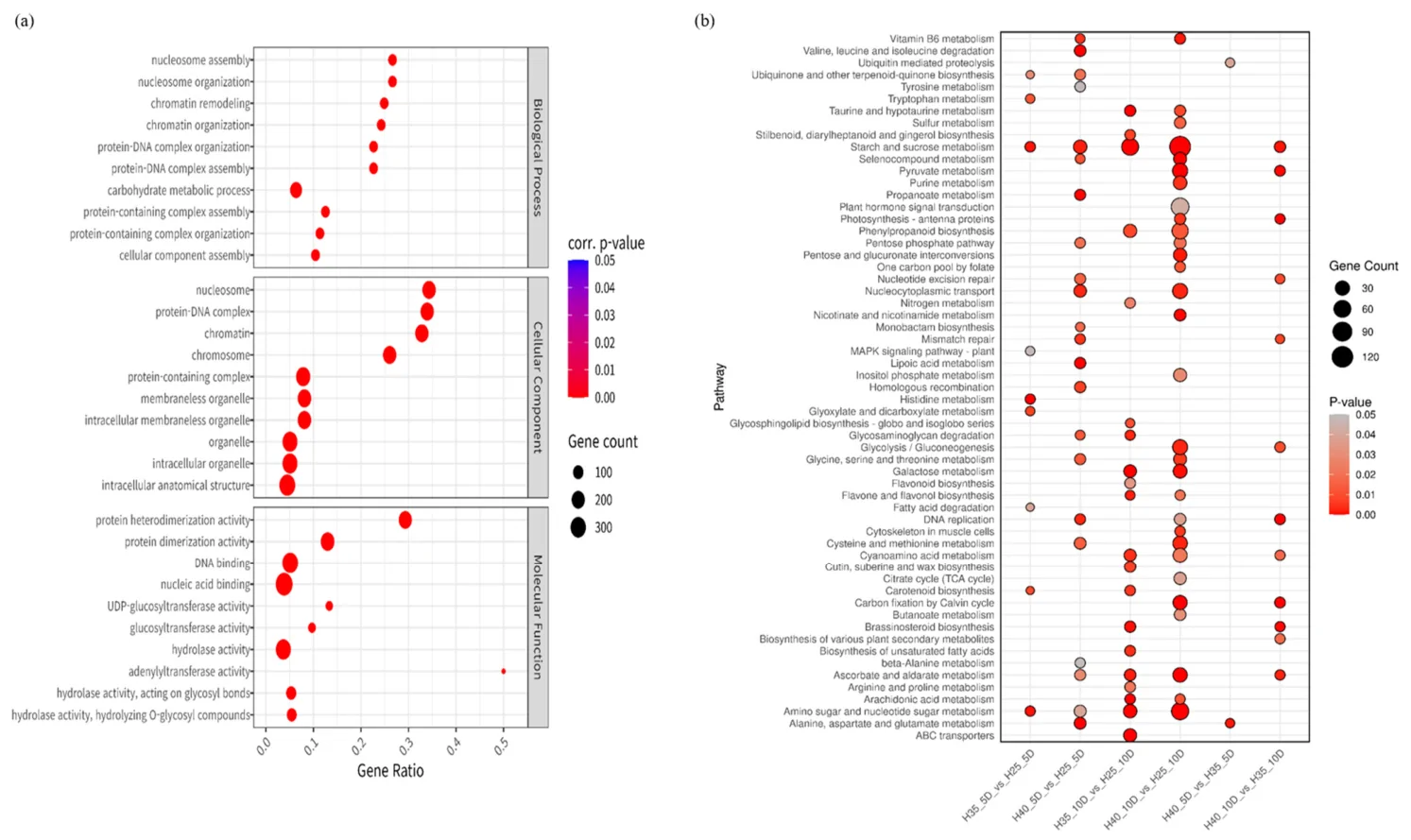
Functional enrichment analysis of down-regulated DEGs in Hwanggeumal (HGA) under heat treatment. (**a**) Gene Ontology (GO) enrichment analysis of down-regulated DEGs identified in the H40_10D versus H25_10D comparison. Enriched GO terms are classified into biological process, cellular component, and molecular function categories. The x-axis represents the gene ratio, bubble size indicates the number of genes associated with each GO term, and bubble color represents the corrected *p*-value. (**b**) KEGG pathway enrichment analysis of down-regulated DEGs across temperature and treatment-duration comparisons in HGA. The x-axis represents the comparison groups, the y-axis represents enriched pathways, bubble size indicates the number of genes associated with each pathway, and bubble color represents the *p*-value.

In Hwanggeumal after 10 days of treatment at 40 °C, downregulated DEGs were mainly associated with nucleosome assembly, chromatin remodeling, and carbohydrate metabolic processes in the GO analysis (Figure 9a). KEGG analysis of downregulated DEGs in Hwanggeumal showed no pathway commonly enriched across the high-temperature-versus-control comparisons, whereas cyanoamino acid metabolism was specifically enriched under the 10-day treatment (Figure 9b).

The corresponding GO enrichment results for down-regulated DEGs in Saekeumkang and up-regulated DEGs in Hwanggeumal are shown in Figure S3 and Figure S4, respectively. Additional KEGG enrichment analyses are shown in Figure S1 and Figure S2.

**Figure 10:**
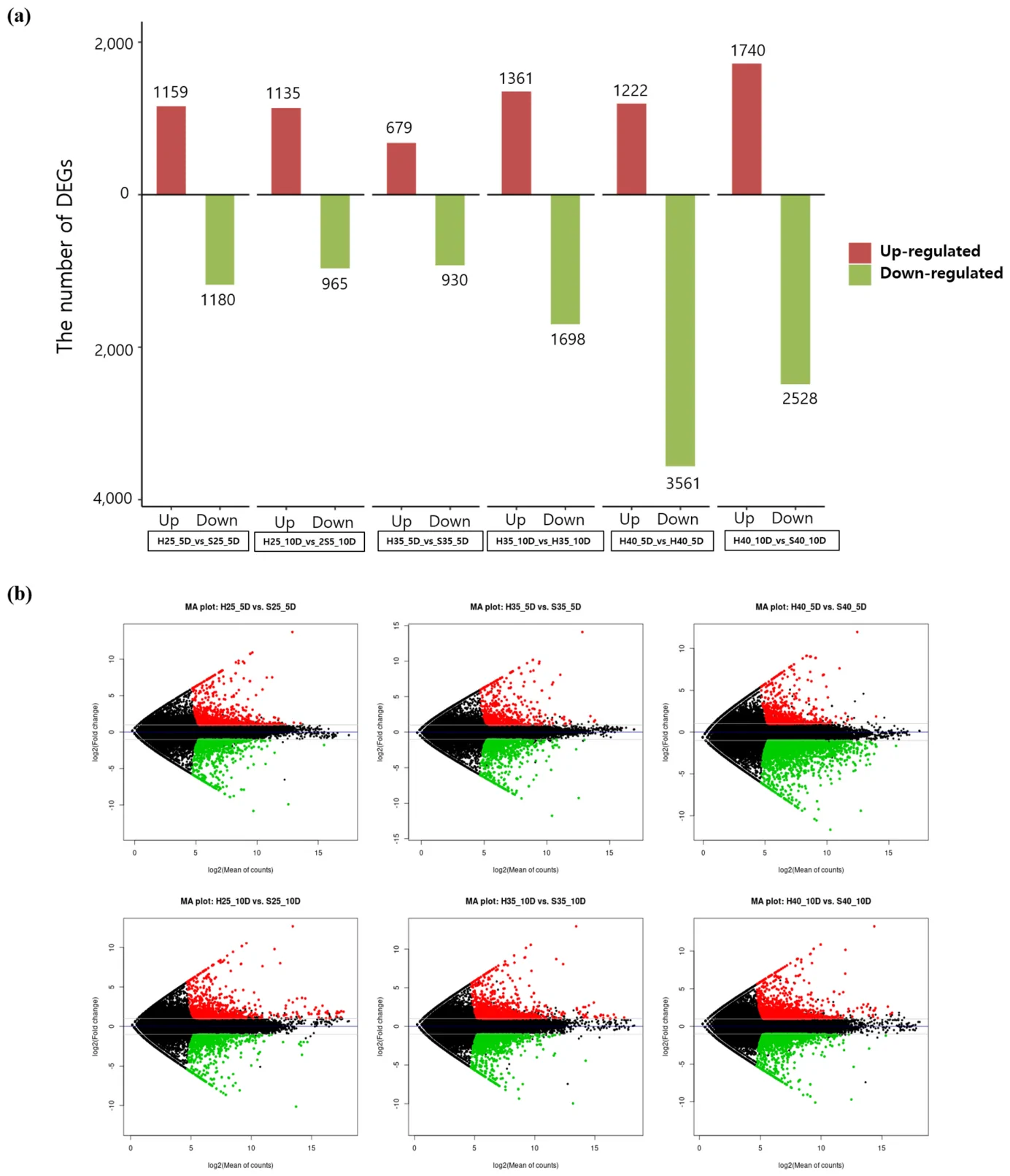
Differentially expressed genes between Hwanggeumal (HGA) and Saekeumkang (SKK) under different temperature and treatment-duration conditions. (**a**) Numbers of up- and down-regulated differentially expressed genes (DEGs) between HGA and SKK at 25, 35, and 40 °C after 5 and 10 days of treatment. (**b**) Corresponding MA plots for each cultivar comparison. Comparisons were H25_5D versus S25_5D, H25_10D versus S25_10D, H35_5D versus S35_5D, H35_10D versus S35_10D, H40_5D versus S40_5D, and H40_10D versus S40_10D. Red and green indicate genes with higher and lower expression in HGA relative to SKK, respectively. Black points in the MA plots indicate non-DEGs.

Direct comparison between Hwanggeumal and Saekeumkang under the same temperature and treatment-duration conditions showed that the number and distribution of differentially expressed genes varied depending on temperature and treatment duration (Figure 10). These results indicate that the transcriptional responses of the two cultivars differed under the same environmental conditions.

## 3. Discussion

### 3.1. Grain Developmental Responses to High-Temperature Treatment at Different Grain-Filling Stages

During the wheat grain-filling process, endosperm formation and expansion, storage compound accumulation, and embryo development proceed continuously; therefore, the effects of high temperature may vary depending on the timing of exposure [11,12]. In this study, the extent of changes in grain area, length, and width in response to high temperature varied depending on treatment timing and cultivar (Figure 1 and Figure 2), indicating that sensitivity to high temperature is not constant throughout the grain-filling process. The significant stage × temperature interactions observed for grain area, width, and length further indicate that the effects of high temperature depended on the developmental stage at which the treatment was imposed. During the early grain-filling period, endosperm cell proliferation and potential grain size are determined, whereas thereafter, cell expansion and storage compound accumulation become increasingly important. Previous studies have reported that high temperatures during grain development can limit endosperm cell number and grain expansion, with the magnitude of these effects varying according to treatment timing, duration, and genotype [11,13,14,15]. Therefore, the stage-dependent differences in grain morphological responses observed in this study can be understood not only in terms of heat intensity but also in relation to the grain developmental processes occurring at the time of treatment.

Endosperm and embryo development also did not always change in the same pattern as external grain morphology (Figure 3). Final grain size is determined through the combined development of external tissues, including the seed coat, as well as the endosperm and embryo [16,17], and changes in overall grain size may not directly reflect the degree of growth of a particular internal tissue. A previous study [11] suggested that partial compensatory responses may occur through subsequent cell expansion when endosperm cell formation is limited by high temperature during early grain development, providing one possible explanation for such non-synchronous responses. This interpretation was also supported by the PCA, in which grain morphological traits and endosperm- and embryo-related traits contributed to the overall variation in different directions, with differences in distribution according to cultivar and temperature (Figure 4). These findings suggest that grain developmental responses to high temperature may result from complex changes involving multiple external and internal developmental traits rather than from changes in a single trait.

### 3.2. Changes in Grain Weight, Protein Concentration, and Storage Metabolism Under High-Temperature Conditions

High temperatures during grain filling affected not only grain development but also 100-grain weight, and the response varied depending on treatment timing and cultivar (Table 1). Final grain weight results from grain expansion and dry matter accumulation during the grain-filling period, and these processes can be affected to varying degrees by high temperature [14]. Previous studies have shown that post-anthesis or short-term high-temperature stress can shorten the grain-filling period and reduce grain width, grain-filling rate, final grain weight, and starch accumulation [18,19,20]. Considering these studies, the changes in 100-grain weight observed in this study can be regarded as the combined result of changes in grain growth and storage compound accumulation in response to high temperature. The fact that the response of 100-grain weight to different treatment timings was not identical between the two cultivars also indicates that these effects may vary not only with heat intensity but also with the developmental stage at which the plants were exposed and their genetic background.

Grain protein concentration responded differently from 100-grain weight, and differences according to treatment timing were observed between Saekeumkang and Hwanggeumal (Table 1). Even if grain protein concentration increases under high-temperature conditions, it is difficult to directly interpret this as an increase in absolute protein accumulation. If the reduction in grain dry weight and starch accumulation is greater than the reduction in protein accumulation, the relative protein concentration may increase, and changes in the spatial distribution of grain protein due to high temperature have also been reported [21]. Previous studies have also shown that high temperature can alter grain dry mass, protein concentration, and the accumulation and composition of storage proteins, with the extent of these changes varying among genotypes and treatment timings [19,22,23,24,25]. Therefore, the protein concentration response observed in this study should not be interpreted merely as a simple effect of high temperature, but rather in the context of grain dry matter accumulation, storage protein formation, treatment timing, and cultivar.

Along with these changes in storage compound accumulation, the expression of genes related to starch and storage protein synthesis also varied with treatment temperature and elapsed time (Figure 5 and Figure 6). TaSSI, TaGBSSI, and TaSBEII are involved in different steps of starch biosynthesis; high temperatures during early grain filling have been reported to alter starch accumulation and the expression of genes associated with carbohydrate and starch metabolism [19,20,26]. In particular, transcriptome analysis of developing wheat grains has also revealed changes in the expression of genes associated with starch biosynthetic pathways in response to high temperature [26]. Therefore, the temporal responses of starch biosynthesis-related genes observed in this study suggest that storage carbohydrate accumulation during grain filling may be regulated at the transcriptional level by high temperature. However, because changes at the transcriptome or RT-qPCR level alone cannot directly determine actual enzyme activity or metabolic flux, these results should be interpreted as molecular evidence for the potential regulation of starch metabolism.

The expression of the storage protein-related genes α-Gli, g1Gli, Dx2, and Dy12 also showed different patterns depending on treatment timing and elapsed time (Figure 5 and Figure 6), whereas final grain protein concentration did not always change in the same direction (Table 1). RT-qPCR results reflect the transcriptional response of specific storage protein genes at particular time points, whereas final protein concentration reflects the relative amount of total protein accumulated during grain filling in relation to total grain dry weight. High temperature can also alter the composition and polymerization of gliadin and glutenin and may affect grain protein concentration differently from absolute protein accumulation [22,23,25]. These findings support the interpretation that the transcriptional response of a specific storage protein gene and final grain protein concentration should not be regarded as equivalent indicators.

### 3.3. Transcriptomic Responses to High-Temperature Intensity and Duration

In the RNA-seq analysis, the scale of transcriptomic changes varied depending on the intensity and duration of high-temperature exposure, and these responses were not identical between Saekeumkang and Hwanggeumal (Figure 7). Previous transcriptomic studies of developing wheat grains have also reported that high temperature induces numerous DEGs and regulates a wide range of functional groups, including signal transduction, starch metabolism, antioxidant responses, and stress-response pathways [26,27]. Therefore, the results of this study, in which more DEGs were identified at higher temperatures, indicate that the extent of transcriptional reprogramming may increase with increasing stress intensity. However, the total number of DEGs alone does not indicate the biological significance of the heat response, and it is necessary to consider which functional gene groups are differentially regulated.

In Saekeumkang subjected to 40 °C for 5 days, the upregulated DEGs were enriched in protein folding, protein maturation, and chaperone-related functions (Figure 8). An increase in abnormally folded proteins at high temperatures activates the HSF–HSP system and molecular chaperones, which play roles in protein stabilization, refolding, and quality control. Previous studies have also shown heat-responsive regulation of HSF/HSP genes and stress-related proteins in wheat [28,29,30]. These results support the interpretation that the protein folding- and chaperone-related GO enrichment observed in this study represents a transcriptional response associated with the maintenance of protein homeostasis under high-temperature conditions. KEGG analysis of upregulated DEGs also showed enrichment of protein processing in the endoplasmic reticulum across high-temperature comparisons (Figure 8b), further supporting the involvement of protein quality-control processes in the heat response. Meanwhile, in Hwanggeumal subjected to 40 °C for 10 days, the downregulated DEGs were enriched in functional groups associated with chromatin organization and carbohydrate metabolic processes (Figure 9). This suggests that prolonged exposure to high temperatures can affect not only direct heat-stress responses but also fundamental cellular processes, including transcriptional regulation and storage carbohydrate metabolism. Previous transcriptomic studies of developing grains have similarly reported changes in starch biosynthetic pathways and primary metabolic processes under high-temperature conditions [20,26,27]. KEGG analysis of downregulated DEGs showed treatment-duration-dependent pathway enrichment, including cyanoamino acid metabolism under the 10-day treatment (Figure 9b). However, because GO enrichment analysis indicates the overrepresentation of DEGs associated with specific functional categories, it should not be interpreted as directly demonstrating an actual increase or decrease in the activity of a particular metabolic pathway. In addition, because Figure 8 and Figure 9 were obtained from different cultivars and treatment durations, the two results should not be regarded as a direct comparison between cultivars under the same conditions.

### 3.4. Cultivar-Specific Transcriptomic Responses of Saekeumkang and Hwanggeumal

Direct comparison between Hwanggeumal and Saekeumkang under the same temperature and treatment-duration conditions showed that the number and distribution of differentially expressed genes between the two cultivars varied depending on temperature and treatment duration (Figure 10). Previous studies have likewise reported genotype-dependent variation in heat responses, including differences related to treatment timing and duration, grain mass and quality traits, and grain proteomic responses [15,23,30]. These findings support the interpretation that wheat cultivars can exhibit distinct molecular responses even when exposed to the same high-temperature conditions.

However, caution is needed when interpreting differences in the number of DEGs between cultivars, because transcriptional responsiveness alone does not directly indicate the biological performance of a cultivar under high-temperature conditions. Although numerous heat-responsive genes have been identified in transcriptomic studies comparing wheat genotypes during grain filling [31], differences in DEG number should be interpreted in the context of genotype-specific transcriptional responses. Therefore, the differences observed between Saekeumkang and Hwanggeumal in this study should be interpreted as cultivar-specific transcriptional responses to high-temperature conditions. Because RNA-seq treatments were initiated only at 10 DAH, the 5D and 10D comparisons reflect differences in exposure duration during early grain filling and should be interpreted separately from the phenotypic and RT-qPCR analyses conducted at both 10 and 20 DAH. Transcriptomic profiling was restricted to 10 DAH to focus on molecular responses during early grain filling; consequently, the lack of transcriptomic data for 20 DAH limits direct molecular comparison between the two developmental stages.

## 4. Materials and Methods

### 4.1. Plant Materials and Growth Conditions

Two Korean bread wheat (*Triticum aestivum* L.) cultivars, Saekeumkang (SKK) and Hwanggeumal (HGA), were selected from among major wheat cultivars cultivated in Korea. The two cultivars differ in grain quality- and starch-related characteristics and were used to investigate cultivar-specific responses to high-temperature stress during grain filling [32,33]. Seeds used in this study were propagated in 2024 at the National Institute of Crop Science, Jeonju, Republic of Korea. Seeds were maintained at 8 °C for 3 d and subsequently transferred to 22 °C to induce germination and seedling emergence. The seedlings were then vernalized at 8 °C for 3 weeks. After vernalization, individual plants were transplanted into 10-cm-diameter pots containing a 1:1 (*v*/*v*) mixture of potting substrate and paddy-rice nursery substrate, with one plant grown per pot. Plants were maintained under controlled-environment conditions with diurnally varying temperatures ranging from 15 to 22 °C and a photosynthetic photon flux density (PPFD) of approximately 100 μmol m^−2^ s^−1^. No additional fertilizer was applied during the growing period, and plants were irrigated as needed to avoid water limitation. The heading date of each plant was recorded individually and used as the reference point for subsequent heat treatments. The experiment was conducted with three biological replicates per treatment.

### 4.2. Heat Treatments During Grain Filling

Heat treatments were imposed at two developmental stages, 10 and 20 days after heading (DAH), to evaluate stage-dependent responses to high temperature during grain filling. The temperature regimes were established with daily maximum temperatures of 25, 35, and 40 °C and a diurnal temperature range of 10 °C, corresponding to maximum/minimum temperature regimes of 25/15, 35/25, and 40/30 °C, respectively. Plants exposed to the 25/15 °C regime served as the control, whereas those exposed to the 35/25 and 40/30 °C regimes were designated as the heat-treatment groups. Heat treatments were initiated at either 10 or 20 DAH, based on the recorded heading date of each plant, and were maintained for 5 d. For the RT-qPCR time-course analysis, plants were returned to the control temperature regime (25/15 °C) after the 5-d heat treatment and maintained under these conditions until the designated sampling times. All treatments were conducted under a 14 h light/10 h dark photoperiod. Each treatment consisted of three biological replicates, with one plant per replicate.

### 4.3. Grain Sampling and Morphological Analysis

Grain samples used for morphological and internal developmental analyses were collected at harvest following the heat treatments imposed at 10 or 20 DAH. Grain morphological traits were analyzed using Seed 1.0.9, an image-based crop phenotyping analysis program developed through the Digital Breeding Technology Development Program (No. 322065-3) of the Korea Institute of Planning and Evaluation for Technology in Food, Agriculture and Forestry (IPET). For each treatment, grains were randomly selected and arranged with the crease facing downward, and images were captured vertically from above using a camera. The acquired images were binarized and subsequently analyzed using Seed 1.0.9 [34]. A total of 30 grains were analyzed for each treatment across three biological replicates. Grain-level measurements were averaged within each biological replicate, and the replicate means were used as independent observations for statistical analysis (*n* = 3).

Grain area (mm^2^), width (mm), and length (mm) were determined from the processed images. To evaluate embryo and endosperm development, the uppermost point of the embryo was manually identified along the longitudinal axis of each grain image. Based on this reference point, total grain length (*H*), endosperm length (*L*_1_), and embryo length (*L*_2_) were determined. Endosperm and embryo ratios were calculated using the following equations:

(1) $$\mathrm{Endosperm}\;\mathrm{ratio}=\frac{L_{1}}{H}\times 100$$

(2) $$\mathrm{Embryo}\;\mathrm{ratio}=\frac{L_{2}}{H}\times 100$$

where *H* represents total grain length, *L*_1_ represents endosperm length, and *L*_2_ represents embryo length.

### 4.4. Determination of 100-Grain Weight and Protein Concentration

Grain weight and protein concentration were determined using grains collected at harvest from Saekeumkang and Hwanggeumal following the control and heat treatments imposed at 10 and 20 DAH. For 100-grain weight determination, 100 grains from each biological replicate were weighed, and the results were expressed as 100-grain weight (g). Three biological replicates were analyzed for each treatment.

For protein analysis, grain samples were ground prior to measurement. Grain protein concentration was determined using Fourier-transform near-infrared (FT-NIR) spectroscopy with a TANGO II FT-NIR spectrometer (Bruker Optics GmbH & Co. KG, Ettlingen, Germany). Protein concentration was measured independently for each of three biological replicates and expressed as a percentage (%).

### 4.5. RNA Isolation and Quantitative Real-Time PCR Analysis

Developing grain samples were collected at designated time points after the initiation of heat treatment, immediately frozen in liquid nitrogen, and stored at −80 °C until RNA extraction. Total RNA for RT-qPCR analysis was isolated from frozen grain samples using an RNeasy Plant Mini Kit (QIAGEN GmbH, Hilden, Germany) according to the manufacturer’s instructions. Quantitative real-time reverse transcription PCR (RT-qPCR) was carried out using a TB Green^®^ PrimeScript™ RT-PCR Kit II (Takara Bio Inc., Kusatsu, Shiga, Japan) with a CFX96™ Real-Time PCR Detection System (Bio-Rad Laboratories, Inc., Hercules, CA, USA).

The expression of starch biosynthesis-related genes, including TaSSI, TaSBEII, and TaGBSSI, and storage protein-related genes, including α-Gli, g1Gli, Dx2, and Dy12, was examined. Relative gene expression was calculated using the 2^−ΔΔCt^ method, with ACT used as the reference gene for normalization. Each RT-qPCR assay was performed in duplicate using RNA obtained from each biological replicate, and three independent biological replicates were analyzed for each treatment [35]. Primer sequences used for RT-qPCR analysis are provided in Supplemental Table S2.

### 4.6. RNA Sequencing and Transcriptome Analysis

For RNA-seq analysis, a separate heat-treatment experiment was conducted. Heat treatments were initiated at 10 DAH, and plants were continuously exposed to 25/15, 35/25, or 40/30 °C (day/night) for either 5 or 10 d. Developing grains were collected immediately at the end of each treatment period for RNA extraction. Samples of Saekeumkang and Hwanggeumal exposed to 25, 35, and 40 °C were used for RNA sequencing. Following collection, grain samples were stored at −80 °C until RNA preparation. Frozen tissues were ground under liquid nitrogen, and approximately 100 mg of each sample was used for total RNA extraction with TRIzol™ Reagent (Invitrogen, Carlsbad, CA, USA). Residual genomic DNA was removed by DNase I treatment (Qiagen). RNA concentration was quantified using Quant-iT RiboGreen (Invitrogen), and RNA quality was evaluated using RNA ScreenTape on a TapeStation system (Agilent Technologies, Inc., Santa Clara, CA, USA). Samples with an RNA integrity number (RIN) ≥ 6.0 were used for library construction.

RNA-seq libraries were prepared from 1 μg of total RNA using the NEXTFLEX^®^ Rapid Directional RNA-Seq Kit 2.0 (PerkinElmer, Inc., Waltham, MA, USA). Poly(A)-containing mRNA was enriched using poly-T-conjugated beads and fragmented before first- and second-strand cDNA synthesis. Following adenylation and adapter ligation, the resulting libraries were assessed using D1000 ScreenTape (Agilent Technologies). Sequencing was carried out on an Illumina NovaSeq platform (Illumina, Inc., San Diego, CA, USA) in paired-end mode with 2 × 151-bp reads.

Raw sequence reads were processed using Trimmomatic v0.39 to remove adapter sequences and low-quality bases. Quality trimming was performed using a sliding window of four bases with a mean quality score ≥ 15; leading and trailing bases with quality scores below 3 were removed, and reads shorter than 36 bp were discarded. The IWGSC RefSeq v2.1 assembly of Triticum aestivum was used as the reference genome, and the processed reads were aligned to the reference using Bowtie2 (v2.4.1). The average mapping rate across all samples was 84.88%. Gene-level read counts were normalized using DESeq v1.38.0 in R version 3.6.3.

Differentially expressed genes (DEGs) were identified based on a minimum two-fold difference in expression and a false discovery rate (FDR)-adjusted *p*-value ≤ 0.01. Genes with log2 fold-change values > 1 and <−1 were classified as up- and down-regulated, respectively. Hierarchical clustering was applied to the selected DEGs to examine similarities in transcriptional responses among temperature treatments. Gene Ontology (GO) analysis was subsequently used to classify gene functions into biological process, cellular component, and molecular function categories, whereas Kyoto Encyclopedia of Genes and Genomes (KEGG) analysis was conducted to examine enriched metabolic and regulatory pathways.

Three independent biological replicates (*n* = 3) were used for each treatment condition, with one RNA-seq library generated from each biological replicate.

### 4.7. Statistical Analysis

Statistical analyses were performed using R software version 4.2.2. Principal component analysis (PCA) was performed using grain morphological and internal developmental traits to evaluate overall variation among cultivars and temperature treatments. Data are presented as the mean ± standard error (SE) of three biological replicates unless otherwise specified. For grain morphological and internal developmental traits, grain-level measurements were averaged within each biological replicate, and the replicate means were used for statistical analysis (*n* = 3). Three-way ANOVA was performed to evaluate the effects of cultivar, developmental stage, temperature, and their interactions. Differences among treatment groups were evaluated separately for each cultivar using one-way analysis of variance (ANOVA). When significant differences were detected, mean comparisons were conducted using either Tukey’s honestly significant difference (HSD) test or Duncan’s multiple range test, as appropriate, at *p* < 0.05. The specific post hoc test used is indicated in the corresponding figure legends.

## 5. Conclusions

In this study, we compared grain development, 100-grain weight, protein concentration, the expression of genes related to storage compound synthesis, and transcriptomic responses in the Korean wheat cultivars Saekeumkang and Hwanggeumal exposed to high temperatures at different stages of grain filling. Changes in grain area, length, and width, as well as endosperm and embryo development, in response to high temperature varied depending on treatment timing and cultivar, and 100-grain weight and grain protein concentration also showed different responses. In addition, the expression of genes involved in starch and storage protein synthesis varied depending on the timing of high-temperature treatment and the elapsed time after treatment, indicating that storage compound accumulation during grain filling may be temporally regulated in response to high temperature.

Transcriptome analysis showed that the number and functional characteristics of differentially expressed genes varied according to heat intensity and exposure duration, and that gene groups related to protein folding and quality control, chromatin-related processes, and carbohydrate metabolism were associated with the heat responses. Furthermore, different transcriptomic responses were observed between Saekeumkang and Hwanggeumal under the same treatment conditions, indicating that molecular responses to high temperature during grain filling may vary depending on the genetic background of the cultivars. These results suggest that, when evaluating heat responses in wheat during grain filling, not only the intensity of high temperature but also treatment timing, exposure duration, and cultivar should be considered. However, transcriptomic profiling was limited to treatments initiated at 10 DAH, which restricts direct molecular comparison between early and later grain-filling stages. In addition, representative RNA-seq-derived DEGs were not independently validated by RT-qPCR; therefore, future studies should extend transcriptomic analyses to later grain-filling stages and validate key heat-responsive DEGs.

## Figures and Tables

**Table 1:** Effects of heat treatment at different grain-filling stages on 100-grain weight and protein concentration in Saekeumkang and Hwanggeumal.

Cultivar	Treatment Stage	Temperature (°C)	100-Grain Weight (g)	Protein Concentration (%)
Saekeumkang	10 DAH	25	4.45 ± 0.11 ^a^	9.75 ± 0.10 ^c^
		35	4.16 ± 0.06 ^a^	13.72 ± 0.53 ^b^
		40	2.42 ± 0.84 ^b^	16.24 ± 0.12 ^a^
	20 DAH	25	4.45 ± 0.11 ^a^	9.75 ± 0.10 ^c^
		35	3.97 ± 0.21 ^b^	10.81 ± 0.31 ^b^
		40	3.77 ± 0.11 ^b^	11.89 ± 0.19 ^a^
Hwanggeumal	10 DAH	25	4.92 ± 0.06 ^a^	16.74 ± 0.79 ^b^
		35	4.14 ± 0.12 ^b^	16.95 ± 0.03 ^ab^
		40	3.25 ± 0.43 ^c^	18.54 ± 0.93 ^a^
	20 DAH	25	4.92 ± 0.06 ^a^	16.74 ± 0.79 ^a^
		35	4.31 ± 0.08 ^b^	14.74 ± 0.21 ^b^
		40	3.97 ± 0.05 ^c^	14.66 ± 0.10 ^b^

Values are presented as mean ± SE. Different letters indicate significant differences among temperature treatments within each cultivar and treatment stage at *p* < 0.05 by Tukey’s test.

## Data Availability

The original contributions presented in this study are included in the article/supplementary material. Further inquiries can be directed to the corresponding author.

## Supplementary Materials

The following supporting information can be downloaded at: https://www.mdpi.com/article/10.3390/plants15203130/s1, Table S1: Three-way ANOVA for grain developmental and quality traits; Table S2: Primer sequences used for RT-qPCR analysis; Figure S1: KEGG pathway enrichment analysis of down-regulated DEGs in Saekeumkang (SKK) after 5 days of heat treatment at 40 °C; Figure S2: KEGG pathway enrichment analysis of up-regulated DEGs in Hwanggeumal (HGA) after 10 days of heat treatment at 40 °C; Figure S3: GO enrichment of down-regulated DEGs in SKK, S40_5D vs. S25_5D; Figure S4: GO enrichment of up-regulated DEGs in HGA, H40_10D vs. H25_10D.

## Abbreviations

The following abbreviations are used in this manuscript:

SKK	Saekeumkang
HGA	Hwanggeumal
DAH	Days after heading
DAT	Days after treatment
RT-qPCR	Quantitative reverse transcription PCR
RNA-seq	RNA sequencing
DEG	Differentially expressed gene
PCA	Principal component analysis
GO	Gene Ontology
FDR	False discovery rate
