# Supplementary material for: Heat Stress Responses During Grain Filling in Two Korean Wheat Cultivars as Affected by Developmental Stage and Exposure Duration
Source: Plants (Basel). 2026 Oct 13;15(20):3130. doi: 10.3390/plants15203130 (PMC13708808; doi:10.3390/plants15203130)
Supplement: Supplementary file 1 [file plants-15-03130-s001.zip › Table S1, S2.pdf]

Table S1. Three-way ANOVA for grain developmental and quality traits

Trait	Cultivar (C)	Stage (S)	Temperature (T)	C × S	C × T	S × T	C × S × T
Grain area	153.83***	26.10***	53.96***	2.87^{ns}	1.59^{ns}	20.63***	1.00^{ns}
Grain width	23.14***	23.39***	67.96***	2.97^{ns}	0.57^{ns}	19.83***	0.74^{ns}
Grain length	611.64***	14.77***	14.90***	1.15^{ns}	1.81^{ns}	7.99**	3.73*
Endosperm length	79.44***	0.16^{ns}	5.21*	1.10^{ns}	1.54^{ns}	1.07^{ns}	1.07^{ns}
Embryo length	61.50***	4.17^{ns}	1.27^{ns}	0.12^{ns}	3.30^{ns}	1.23^{ns}	0.12^{ns}
Endosperm ratio	9.13**	2.24^{ns}	1.03^{ns}	0.19^{ns}	3.02^{ns}	0.56^{ns}	0.05^{ns}
Embryo ratio	8.79**	1.64^{ns}	1.33^{ns}	0.21^{ns}	2.77^{ns}	0.42^{ns}	0.05^{ns}
100-grain weight	15.47***	12.45**	63.57***	0.21^{ns}	1.36^{ns}	12.89***	2.25^{ns}
Protein concentration	785.05***	203.67***	60.77***	1.58^{ns}	74.17***	59.17***	0.44^{ns}

Values are F-statistics from Type III three-way ANOVA. C, cultivar; S, developmental stage; T, temperature. $p < 0.05$; $p < 0.01$; $p < 0.001$; ns, not significant.

Table S2. Primer sequences used for RT-qPCR analysis in this study

Category	Gene	Description	Forward primer (5′–3′)	Reverse primer (5′–3′)
Starch metabolism	*TaSSI*	Soluble starch synthase (SSS)	GTTTCCCACAGAATAACTGCAGGTTGC	GTATGGTCTTTCGTCATGCCTCGC
Starch metabolism	*TaSBEII*	Starch-branching enzyme (SBE)	GCACCAGTATGTTTCACGGAAACATG	GGCATACACGACCGCAGTTCTG
Starch metabolism	*TaGBSSI*	Granule-bound starch synthase (GBSS)	CTCGCCGCCAACTACGACGTC	TGCTCGGGAACTTCTCCTCCAC
Gliadin metabolism	*α-Gli*	α-type gliadin	AGACCTTTCTCATCCTTGCC	TGTACCAATGGAACTTGCTCT
Gliadin metabolism	*γ-Gli*	γ-type gliadin	ACAGGTGAACCCATGCAAGAATTT	TGCATGATGATGGAATGTATGATGG
Glutenin metabolism	*Dx2*	High-molecular-weight glutenin subunit 2	GCAGCAACTCCAACAACGTA	GCCTGGATAGTATGAAACCTG
Glutenin metabolism	*Dy12*	High-molecular-weight glutenin subunit 12	CCCAAGCGTAACTTCTCCTCGG	TGTTGCCCTTGTCCTGGTTC
Reference gene	*ACT*	Actin	GGAGAAGCTCGCTTACGTG	GGGCACCTGAACCTTTCTGA
