# Supplementary material for: Heat Stress Responses During Grain Filling in Two Korean Wheat Cultivars as Affected by Developmental Stage and Exposure Duration
Source: Plants (Basel). 2026 Oct 13;15(20):3130. doi: 10.3390/plants15203130 (PMC13708808; doi:10.3390/plants15203130)
Supplement: Supplementary file 1 [file plants-15-03130-s001.zip › Supplementary Figures.pdf]

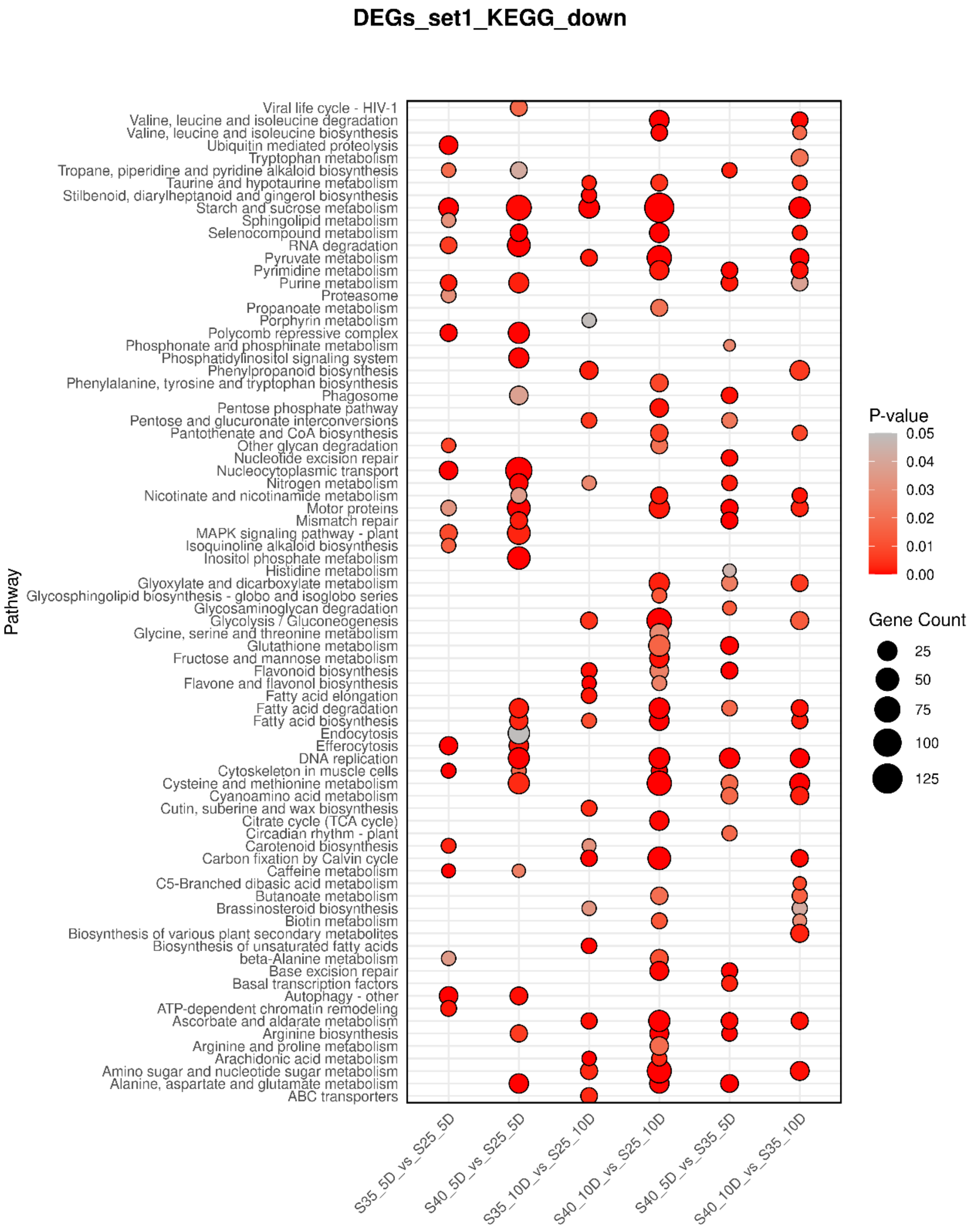

Figure S1. KEGG pathway enrichment analysis of down-regulated DEGs in Saekeumkang (SKK) after 5 days of heat treatment at 40 °C.

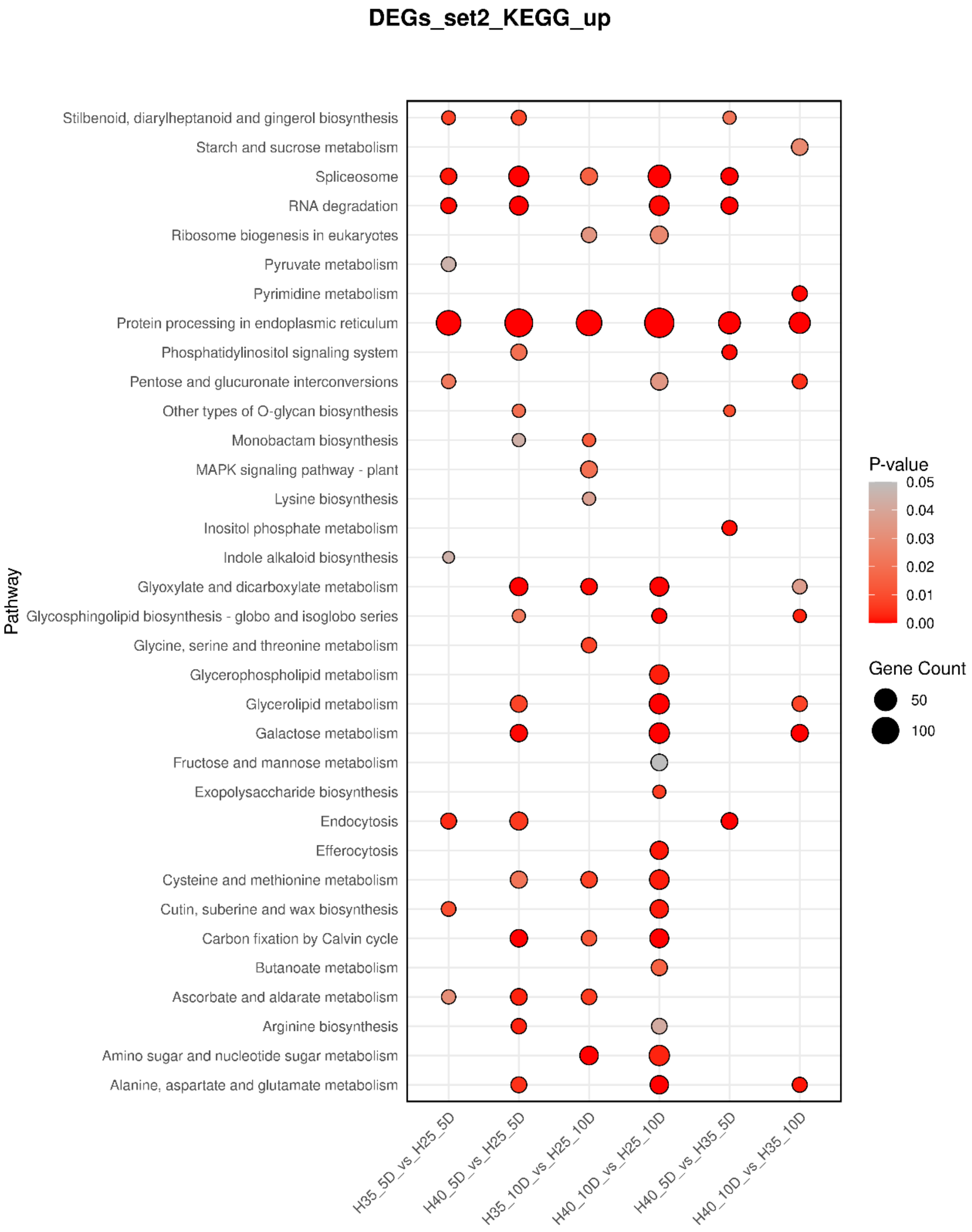

Figure S2. KEGG pathway enrichment analysis of up-regulated DEGs in Hwanggeumal (HGA) after 10 days of heat treatment at 40 °C.

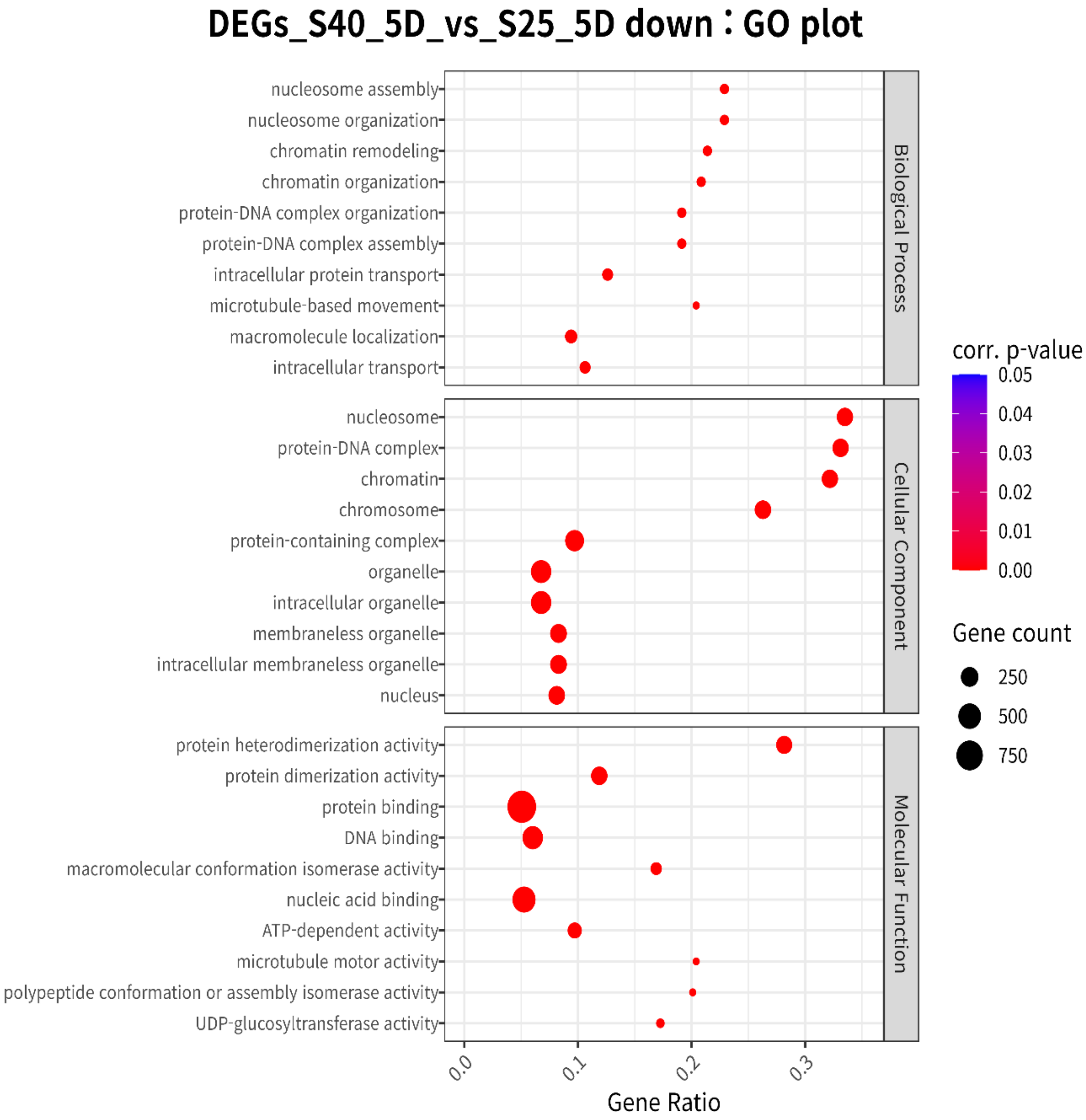

Figure S3. GO enrichment of down-regulated DEGs in SKK, S40_5D vs S25_5D.

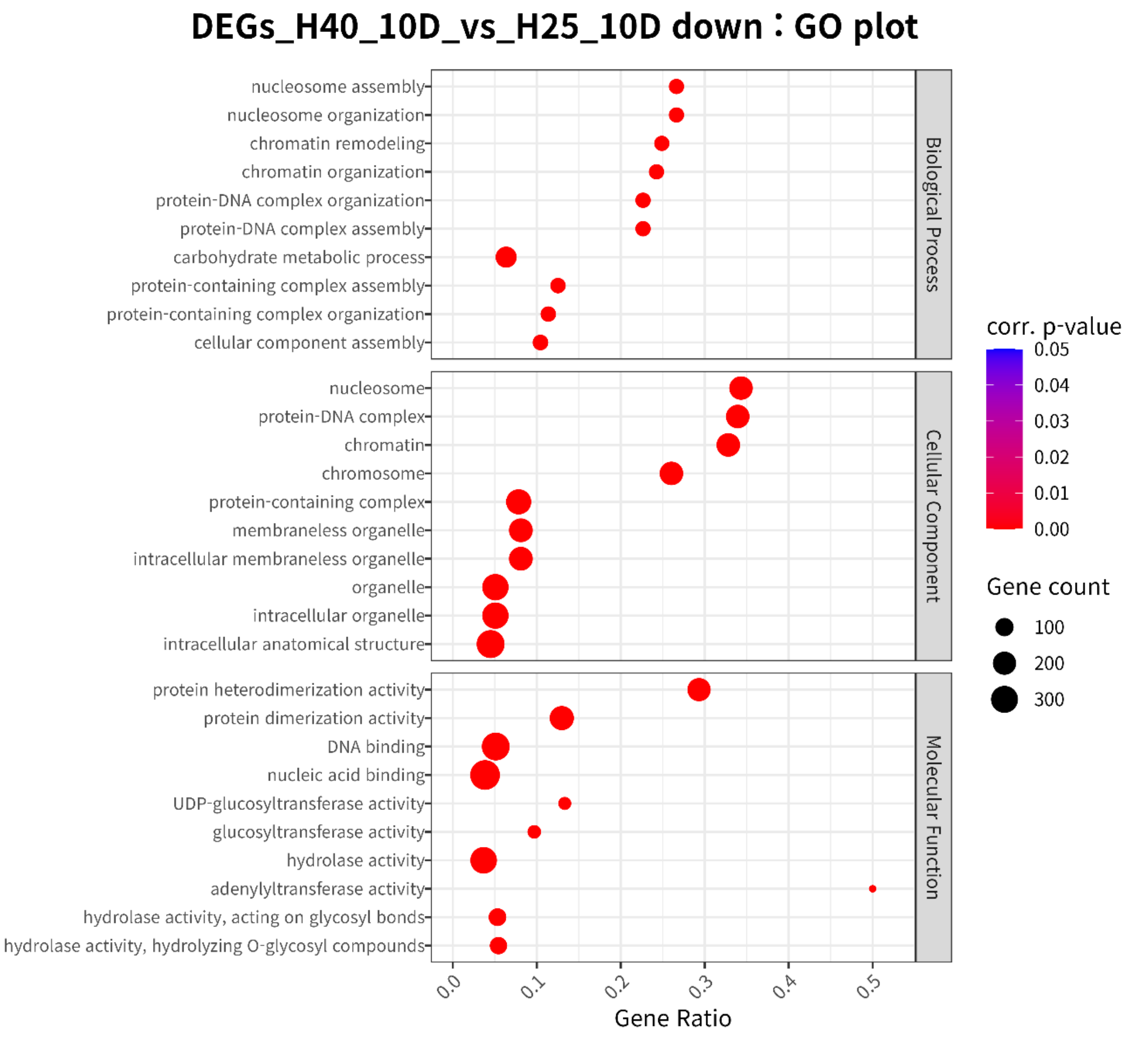

Figure S4. GO enrichment of up-regulated DEGs in HGA, H40_10D vs H25_10D.
